# The CRISPR toolbox in medical mycology: State of the art and perspectives

**DOI:** 10.1371/journal.ppat.1008201

**Published:** 2020-01-16

**Authors:** Florent Morio, Lisa Lombardi, Geraldine Butler

**Affiliations:** 1 School of Biomolecular and Biomedical Science, Conway Institute, University College Dublin, Belfield, Dublin, Ireland; 2 Département de Parasitologie et Mycologie Médicale, Université de Nantes, Nantes Université, EA1155 –IICiMed, Nantes, France; Geisel School of Medicine at Dartmouth, UNITED STATES

## Abstract

Fungal pathogens represent a major human threat affecting more than a billion people worldwide. Invasive infections are on the rise, which is of considerable concern because they are accompanied by an escalation of antifungal resistance. Deciphering the mechanisms underlying virulence traits and drug resistance strongly relies on genetic manipulation techniques such as generating mutant strains carrying specific mutations, or gene deletions. However, these processes have often been time-consuming and cumbersome in fungi due to a number of complications, depending on the species (e.g., diploid genomes, lack of a sexual cycle, low efficiency of transformation and/or homologous recombination, lack of cloning vectors, nonconventional codon usage, and paucity of dominant selectable markers). These issues are increasingly being addressed by applying clustered regularly interspaced short palindromic repeats (CRISPR)–Cas9 mediated genetic manipulation to medically relevant fungi. Here, we summarize the state of the art of CRISPR–Cas9 applications in four major human fungal pathogen lineages: *Candida* spp., *Cryptococcus neoformans*, *Aspergillus fumigatus*, and Mucorales. We highlight the different ways in which CRISPR has been customized to address the critical issues in different species, including different strategies to deliver the CRISPR–Cas9 elements, their transient or permanent expression, use of codon-optimized *CAS9*, and methods of marker recycling and scarless editing. Some approaches facilitate a more efficient use of homology-directed repair in fungi in which nonhomologous end joining is more commonly used to repair double-strand breaks (DSBs). Moreover, we highlight the most promising future perspectives, including gene drives, programmable base editors, and nonediting applications, some of which are currently available only in model fungi but may be adapted for future applications in pathogenic species. Finally, this review discusses how the further evolution of CRISPR technology will allow mycologists to tackle the multifaceted issue of fungal pathogenesis.

## Introduction

The most common fungal diseases are superficial infections, affecting almost two billion people worldwide [1]. Despite being much less common, invasive fungal infections are nonetheless associated with high mortality rates. Fungal pathogens are therefore increasingly recognized as a major human threat. According to current (likely under-) estimates, 1.6 million people die every year from invasive diseases caused by different fungal species (mainly *Cryptococcus*, *Candida*, *Aspergillus*, and *Pneumocystis*), almost the same as from tuberculosis [2]. We are also currently facing a concerning escalation of antifungal resistance, with reports of azole-resistant *Aspergillus fumigatus* and the spread of multidrug-resistant *Candida auris* [3,4]. In this context, there is an urgent need for efficient genetic manipulation tools to further our understanding of the biology and pathophysiology of fungal pathogens and to decipher drug resistance mechanisms, which are critical for developing novel therapeutic strategies. However, genetic manipulation has often been time-consuming and cumbersome in fungi, particularly in species with diploid genomes that lack a sexual cycle. A low efficiency of transformation and/or homologous recombination, the absence of natural plasmids, and the lack of cloning vectors or the limited number of dominant markers available for selection are additional factors hampering this process [5,6].

Over the last decade, the groundbreaking discovery of clustered regularly interspaced short palindromic repeats (CRISPR)-Cas9 revolutionized genome editing [7]. Normally involved in bacterial adaptive immunity against bacteriophages, the discovery that Cas9 endonuclease could not only target infecting viral DNA but virtually any DNA paved the road for the application of this technique to eukaryotic cells, including mammalian cells, plants, and fungi (including the model yeast *Saccharomyces cerevisiae*) [8]. In this review we describe the state of the art of CRISPR–Cas9 applications in four major human fungal pathogens, *Candida* spp., *Cryptococcus neoformans*, *A*. *fumigatus*, and Mucorales, dissecting the customized strategies for the delivery of functional CRISPR–Cas9 elements. Moreover, we discuss the most promising future perspectives and their further development that will allow mycologists to face the challenge of fungal pathogenesis.

## The CRISPR–Cas9 toolbox: How does it work?

The potential of CRISPR-associated protein Cas9 for genome editing was first reported in 2012 [7,9]. Cas9, a type II RNA-guided endonuclease, introduces a double-stranded break (DSB) 3 bp upstream of a protospacer adjacent motif (PAM, NGG for *Streptococcus pyogenes* Cas9). Cas9 is directed to the site by a single-guide RNA (sgRNA), encompassing both target specificity and Cas9 binding activity—mediated in the native bacterial system by the CRISPR-RNA (crRNA) and the *trans*-activating crRNA (tracrRNA), respectively. The DSB can be repaired by nonhomologous end joining (NHEJ), usually resulting in insertions or deletions leading to frame shift of the reading frame and premature stop codons. Microhomology-mediated end joining (MMEJ) is an alternative, also error-prone repair pathway requiring short homology regions (2–40 bp) [10]. If a donor template with appropriate homology regions is available (e.g. the second allele in diploid genomes, or exogenous DNA), repair may occur by homology-directed repair (HDR). Precise editing exploits HDR machinery by providing the cells with donor DNAs harboring the intended mutation flanked by homology arms. Although conventional methods can also be used to introduce mutations by HDR, DSBs significantly increase the rate of these events by up to 4,000-fold [11–15]. As a result, introducing Cas9-mediated DSBs opens up the possibility to exploit HDR even in species in which this pathway is normally not efficient.

## Tips and tricks of CRISPR–Cas9 in fungi

Expression of *CAS9*, codon optimization, and nuclear localization of the protein are crucial for the success of CRISPR–Cas9 gene editing. In addition, the expression of a sgRNA suitable for interaction with Cas9 is pivotal. Not surprisingly, there is no one method for introducing these elements that is suitable for every fungal species. Firstly, whereas the versions of Cas9 with codons optimized for human cells work in *C*. *neoformans* and *A*. *fumigatus* [16,17], further modifications are required in many *Candida* species, in which CTG is translated as serine [18]. The choice of the regulatory elements to drive expression of *CAS9* and sgRNA is another potential concern. Despite some flexibility among closely related species, species-specific promoters are usually more effective [19,20]. The sgRNA is usually expressed from an RNA pol III promoter because it is a nucleus-localized noncoding RNA molecule. Although RNA pol III promoters of some species have been characterized (e.g., *SNR52* promoter in *C*. *albicans*), mining a genome to find de novo RNA pol III promoters is a cumbersome process of trial and error, which can only be partially streamlined if transcriptome data are available to guide the search. For this reason, suitable RNA pol II promoters can be used instead, provided that the sgRNA molecule is flanked by RNA elements (typically ribozymes or tRNAs) that will mediate the removal of the 5’ cap and the 3’ poly(A) tail [21].

An additional level of complexity is introduced by the delivery system chosen. Unlike *S*. *cerevisiae*, no natural plasmids are found in fungal pathogens, and circular vectors are not always stable (e.g., most vectors need to be linearized before being transformed into *C*. *albicans* and *C*. *neoformans*). In *Candida glabrata*, *Candida parapsilosis*, and *A*. *fumigatus*, the availability of autonomously replicating sequences allows the use of episomal plasmids [22,23]. In Mucorales, by contrast, plasmids usually do not integrate, and when they do, they may lead to unexpected genomic changes or recircularization [24]. One way to get around these problems is to use in vitro assembled ribonucleoprotein complexes (RNP) [25–28]. In this approach, cells are directly transformed with the Cas9–sgRNA complex, thus bypassing the need for any species-specific expression or delivery tailoring. However, the higher cost makes this approach unsuitable for large-scale efforts.

Regardless of the way that the DSB is induced, the outcome depends on the repair strategy. Fungal species differ intrinsically in DNA-repair mechanisms. *C*. *albicans* and *C*. *parapsilosis*, like *S*. *cerevisiae*, rely mostly on HDR, whereas *Candida lusitaniae*, *A*. *fumigatus*, and Mucorales rely mostly on NHEJ, and *Candida glabrata* and *Candida tropicalis* use both HDR and NHEJ [16,20,29–31]. Disrupting NHEJ-related genes increases HDR efficiency in some species (e.g., *Ku70*/*Lig4* deletion increases efficiency from 25% to 81% in *C*. *lusitaniae* haploid cells) [20]. However, this strategy is not ideal, because NHEJ-defective strains may display reduced virulence [17]. Even in species with high levels of HDR, CRISPR–Cas9 increases the efficiency of this process so much that shorter sequences can be used to direct homologous recombination. As an example, homology arms as short as 50 bp arms resulted in 61% efficiency for deleting *ADE2* in *C*. *albicans* [30], and homology arms of 30 bp resulted in almost a 100% gene-editing efficiency in *Candida parapsilosis* [29]. Very often *ADE2*, a gene involved in the adenine biosynthetic pathway, is selected as proof of principle target when implementing CRISPR–Cas9 in a new species. In fact, *ade2* mutants are easy to screen because the interruption of the pathway results in the accumulation of a red to pink pigment that colors the colonies.

Finally, general recommendations for guide design to ensure efficiency and reduce off-target effects also apply to these fungi [32]. Numerous guide-design software programs are now available, some allowing uploading of custom genomes, such as EUPaGDT (http://grna.ctegd.uga.edu) or CHOPCHOP (http://chopchop.cbu.uib.no/).

## Current methods available for genome editing in fungal pathogens

**Fig 1:**
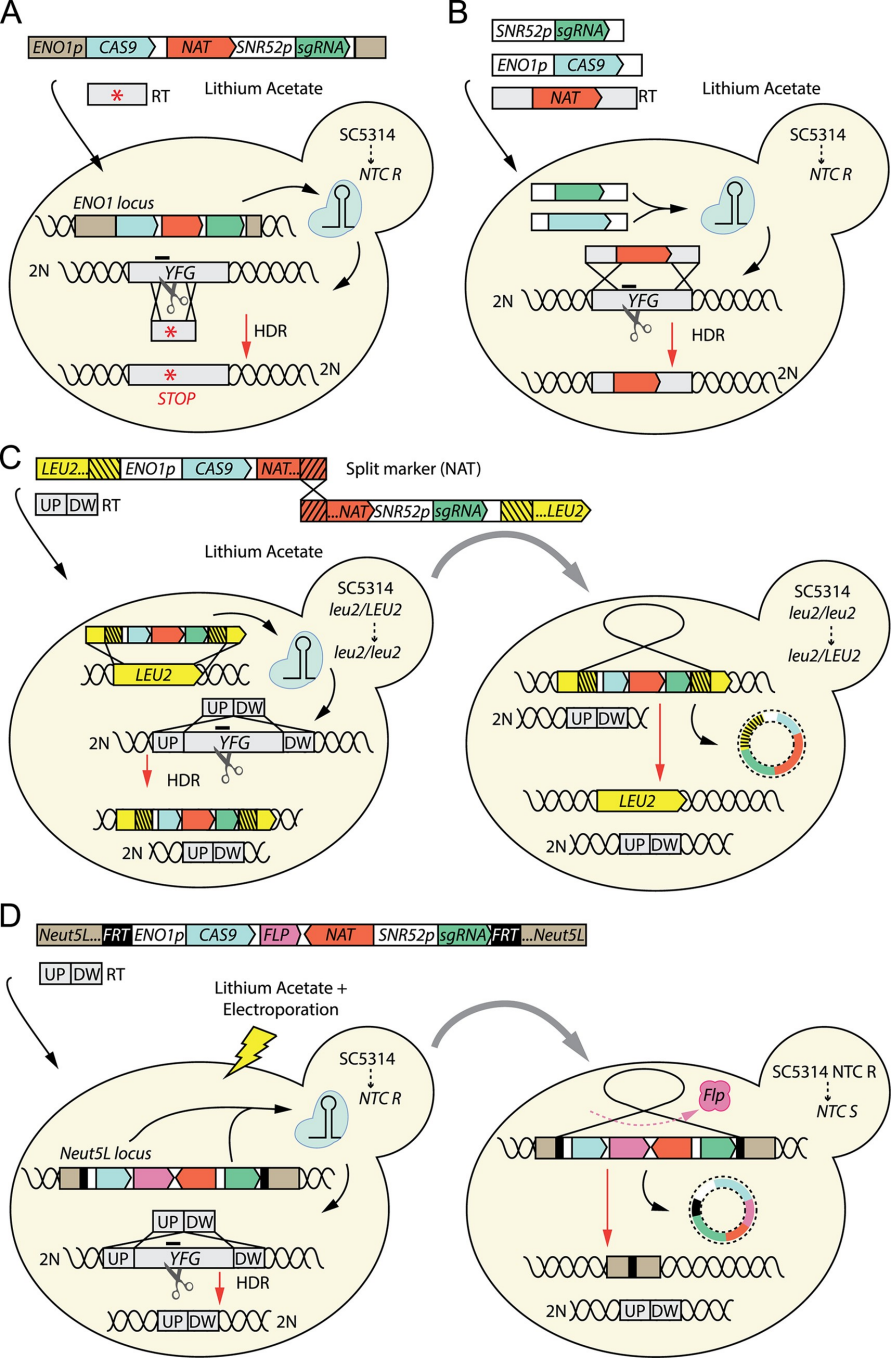
CRISPR–Cas9 methods in *Candida albicans*. The vignettes illustrate four representative methods used for CRISPR–Cas9 genetic manipulation of *C*. *albicans*. The constructs expressing Cas9 and the sgRNA and the RT are depicted. Promoter sequences are in white, unless they constitute part of the upstream homology arm for the integration in the genomic locus, in which case they have the same color as the gene targeted for integration. The transformation method is also indicated. The targeted site on *YFG* is indicated by a dash. The ploidy of the alleles is indicated next to the DNA helix. **(A)** *CAS9*, the sgRNA, and the *NAT* cassette are stably integrated at the *ENO1* locus, and HDR mediates the introduction of an RT carrying a stop codon following Cas9-induced cleavage [18]. **(B)** Transformation of linear DNA fragments into the cell results in transient expression of Cas9 and sgRNA, and HDR results in the integration of a *NAT* cassette into the targeted locus [33]. A further modification of this system allows for marker recycling [34]. **(C)** In the LEUpOUT approach, a split-marker strategy enables the in vivo reconstitution of the selectable cassette for the expression of the CRISPR elements. Integration of the reconstituted cassette disrupts the functional *LEU2* locus of a *LEU2/leu2* heterozygous strain. HDR between the targeted locus and the RT following Cas9 cut results in the deletion of the gene. HDR between directed repeats flanking the cassette (depicted as black lines on yellow background) mediates the removal of CRISPR elements and the restoration of the *LEU2* ORF, which can be selected on leucine dropout medium [35]. Note that the *leu2* deleted allele originally present in the *LEU2/leu2* heterozygous strain remains untouched during the entire procedure, and so it is not depicted in the scheme. **(D)** An improved version of the method depicted in (A) allows for marker recycling. A cassette expressing the CRISPR elements and containing a *FLP* recombinase is flanked by *FRT* sequences (targets of Flp) and homology arms that mediate its integration in the *Neut5L* locus. HDR with an RT results in the target gene deletion. Activation of the inducible promoter driving the expression of Flp is followed by excision of the cassette, leaving only an *FRT* sequence in the *Neut5L* locus [30]. CRISPR, clustered regularly interspaced short palindromic repeats; DW, downstream; HDR, homology-directed repair; NTC R, Nourseothricin resistant; NTC S, Nourseothricin susceptible; ORF, open reading frame; RT, repair template; sgRNA, single-guide RNA; UP, upstream; *YFG*, your favorite gene.

**Fig 2:**
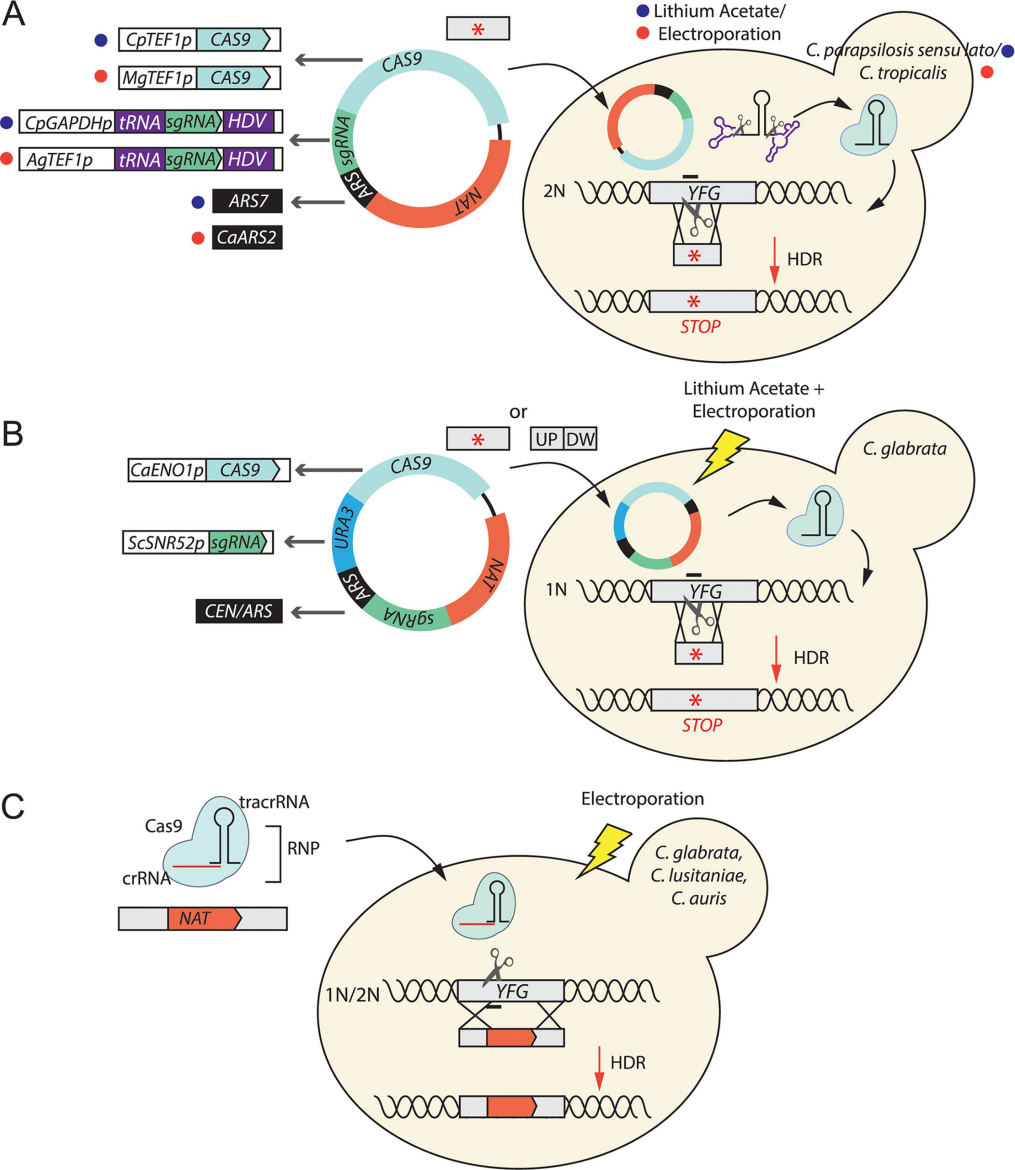
CRISPR–Cas9 methods in non-*albicans Candida* species. The vignettes illustrate representative methods for implementation of CRISPR–Cas9 in non-*albicans* species, including *C*. *parapsilosis sensu lato*, *C*. *tropicalis*, *C*. *glabrata*, *C*. *lusitaniae*, and *C*. *auris*. The ploidy of the species is indicated next to the DNA helices. **(A)** Two different selectable episomal plasmids allow the scarless introduction of premature stop codons by HDR in *C*. *parapsilosis sensu lato* and *C*. *tropicalis*. The relevant species-specific promoters and ARSs are depicted on the left side (blue dot: *C*. *parapsilosis sensu lato*; red dot: *C*. *tropicalis*). The sgRNA is expressed from an RNA pol II promoter, and the release of the mature molecule is mediated by the cleavage of the upstream tRNA molecule and the downstream HDV ribozyme. The plasmids are easily lost in the absence of selection [29]. **(B)** A similar approach can be used for gene editing and deletion in *C*. *glabrata*. In this case, the sgRNA is expressed from the *Saccharomyces cerevisiae* RNA pol III *SNR52* promoter [30]. **(C)** The use of CRISPR RNPs, in which the crRNA and tracrRNA are assembled in vitro with Cas9 and then electroporated into the cells, results in the integration of a *NAT* cassette into the targeted gene in *C*. *glabrata*, *C*. *lusitaniae*, and *C*. *auris* [25]. Although this method does not require species-specific adaptation of the regulatory elements necessary for the expression of the CRISPR elements, marker recycling is not possible. Ag, *Ashbya gossypii*; ARS, autonomously replicating sequence; Ca, *Candida albicans*; Cp, *C*. *parapsilosis*; CRISPR, clustered regularly interspaced short palindromic repeats; crRNA, CRISPR-RNA; DW, downstream; HDR, homology-directed repair; HDV, human hepatitis delta virus; Mg, *Meyerozyma guilliermondii*; RNP, RNA–Cas9 protein complex; Sc, *Saccharomyces cerevisiae*; sgRNA, single-guide RNA; tracrRNA, *trans*-activating RNA; UP, upstream; *YFG*, your favorite gene.

**Fig 3:**
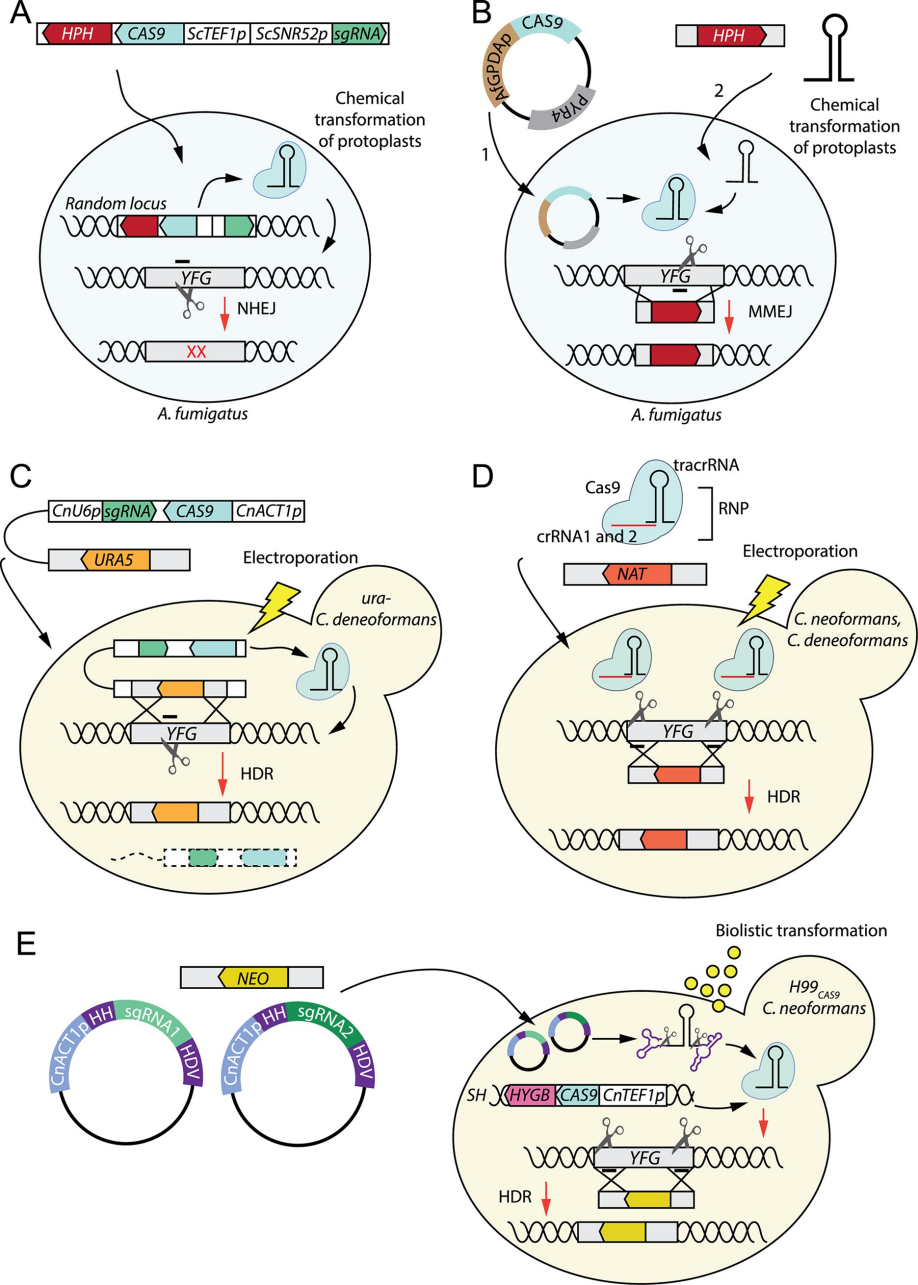
CRISPR–Cas9 methods in *Aspergillus fumigatus*, *Cryptococcus neoformans and C*. *deneoformans*. The vignettes illustrate representative methods for CRISPR–Cas9 genetic manipulation of *A*. *fumigatus* (A–B), *C*. *neoformans*, and *C*. *deneoformans* (C–E). All the species depicted here are haploid. **(A)** In *A*. *fumigatus*, the CRISPR elements are integrated randomly in the genome of protoplasts and selected due to the presence of an *HPH* cassette. NHEJ results in indels at the cleavage site [16]. **(B)** MMEJ can be used to integrate an *HPH* cassette into a desired locus by using short homology arms. The strain is first transformed with a plasmid expressing Cas9 and containing a *PYR4* marker and then with an in vitro transcribed sgRNA and the *HPH* cassette [45]. **(C)** In this “suicide” system, a cassette containing both the CRISPR elements and a *URA5* marker flanked by homology arms to the target site can be transformed into an uracil auxotrophic strain of *C*. *deneoformans*. The double-strand break induced by the transiently expressed Cas9 is repaired by HDR with the *URA5* cassette, and the crossover results in the release of the DNA of the CRISPR elements that induced the cleavage in the first place, which may be degraded (hence the name “suicide” system) [17]. **(D)** An RNP method was developed for *C*. *neoformans* and *C*. *deneoformans*, similar to the one adapted to *Candida* species. The cells are transformed with a mixture of two RNPs, each targeting a different end of the target gene, thus maximizing the chances of gene deletion [27]. **(E)** *CAS9* can be integrated in the *Safe Haven* on *C*. *neoformans* H99 and selected due to the presence of a *HYGB* cassette. This strain is then be transformed with two transient plasmids expressing two sgRNAs flanked by ribozymes from the native *ACT1* promoter, targeting the beginning and the end of the target gene. A *NEO* cassette flanked by homology arms to the target gene is used for HDR [53]. Af, *Aspergillus fumigatus*; Cn, *Cryptococcus neoformans*; CRISPR, clustered regularly interspaced short palindromic repeats; crRNA, CRISPR-RNA; HDR, homology-directed repair; HDV, human hepatitis delta virus; HH, hammerhead; MMEJ, microhomology-mediated end joining; NHEJ, nonhomologous end joining RNP, RNA–Cas9 protein complex; Sc, *Saccharomyces cerevisiae*; sgRNA, single-guide RNA; tracrRNA, trans-activating RNA; *YFG*, your favorite gene.

Figs 1 to 3 illustrate representative methods for CRISPR-genome editing developed for the fungal pathogens discussed below.

### *Candida* species

In 2015, Vyas and colleagues reported the first CRISPR–Cas9 method in *C*. *albicans* [18], which relied on the integration of *CAS9* and sgRNA either as a single cassette at the *ENO1* locus (solo system) or as two separate cassettes at the *ENO1* and *RP10* loci, respectively (duet system). Transformed strains were selected using Nourseothricin, due to the presence of the *NAT* gene in the cassette(s). Expression of a CTG-codon–optimized *CAS9* is driven from the promoter at the integration site, whereas the sgRNA is expressed from the RNA pol III promoter *CaSNR52*. HDR-mediated gene editing was achieved by using a donor DNA with 50 bp homology arms to introduce premature stop codons in the *ADE2* gene, with variable efficiencies (20% to 80%), depending on the vector used (solo or duet) (Fig 1A). The system was also used to simultaneously target members of a gene family with a single sgRNA and disrupt essential genes by generating conditional alleles. However, in the original system, the selection marker could not be recycled, making sequential editing in the same background virtually impossible, and stable *CAS9* integration in the genome raised concerns about potential off-target effects.

Min and colleagues addressed the problem by transiently expressing *CAS9* and the sgRNA in *C*. *albicans* [33]. The repair template contained an *NAT* cassette, which was used to replace the target gene (Fig 1B). Deletion of both alleles occurred either by independent recombination at both or by recombination at one allele followed by allelic gene conversion. Though this method avoided integrating *CAS9*, it was not until the following year that serial mutagenesis in the same background could be achieved [34,35]. Huang and colleagues [34] developed an ingenious marker-recycling model called CRISPR-mediated marker excision (CRIME), in which the selection marker used to delete the gene of interest is flanked by directly repeated sequences. Following the deletion of the target gene, the CRISPR–Cas9 system is used again and induces a DSB into the marker itself, leading to marker excision by recombination between the repeats [34].

Nguyen and colleagues [35] developed alternative strategies to allow marker recycling in different *C*. *albicans* genetic backgrounds, namely the LEUpOUT system—which requires an *LEU2/leu2Δ* parental strain—or the HIS–FLP system, applicable to any strain. In both cases, the transient integration of a cassette containing both a selection marker and *CAS9* and sgRNA at a single locus (either *LEU2* or *HIS*)—allowing the deletion of the gene of interest—is followed by the excision of the cassette by either recombination between direct repeats (LEUpOUT) or activation of an *FLP* recombinase [35] (Fig 1C). Markerless deletion in a single transformation and the absence of a cloning step are the main advantages of this method, as well as the possibility to generate reconstituted strains.

Most *C*. *albicans* editing strategies used an RNA pol III promoter to express the sgRNA. However, Ng and colleagues [21] showed that efficiency of CRISPR–Cas9 in *C*. *albicans* could be further improved by up to 10 times when the sgRNA is expressed from the strong RNA pol II (ADH) promoter and is flanked by an upstream tRNA sequence and a downstream human hepatitis delta virus (HDV) ribozyme to release any added 5’ cap and 3’ poly(A) tail.

Vyas and colleagues [30] also developed a method for marker recycling. The *CAS9*, sgRNA, and *NAT* sequences are integrated at the *NEUT5L* locus and subsequently removed due to the activation of a *FLP* recombinase (Fig 1D). Transformation with a donor DNA juxtaposing 50 bp upstream and downstream of a given *ORF* results in an unmarked deletion mutant. Because the marker selecting for the presence of the CRISPR–Cas9 elements is recyclable and no marker integration occurs at target gene, the system allows the construction of multiple deletion mutants in virtually any genetic background.

In 2017, Shapiro and colleagues [36] adapted the gene-drive principle described in *S*. *cerevisiae* to *C*. *albicans*, thanks to the discovery of mating-competent, *C*. *albicans* haploids [37–38]. Firstly, a cassette expressing *CAS9* and two sgRNAs is integrated at the *NEUT5L* locus in a haploid cell. The sgRNAs are flanked by sequences homologous to the target gene. Following mating, an intact copy of the target gene is introduced. The sgRNAs direct Cas9 to the target gene, introducing DSBs that are repaired by HDR using the *CAS9* and sgRNA cassette. This step will occur in any subsequent mating event, acting as a “drive” allele. When haploid cells containing the drive allele are mated to wild-type cells, the incoming wild-type allele is cut by Cas9 and deleted by replacement with the sgRNA module via HDR. Mating of haploid *Candida* cells deleted for specific *ORFs* therefore rapidly leads to homozygous diploid double-deletion mutants. This platform allows the analysis of genetic interaction by combining deletions of two genes in the same background. However, the stable integration of *CAS9* within the genome requires monitoring of possible off-target effects.

CRISPR–Cas9 methods have also been implemented in non-*albicans Candida* species. Like *C*. *albicans*, the *C*. *parapsilosis sensu lato* complex species, *C tropicalis*, *C*. *lusitaniae*, and *C*. *auris* are members of the CTG clade. Norton and colleagues developed the first transient CRISPR–Cas9 expression system for gene deletion in the genetically intractable *C*. *lusitaniae* [20]. Editing efficiencies of 36% were achieved using 1 kb homology arms to direct integration of a *NAT* cassette at the target gene, using *C*. *lusitaniae*-specific promoters to express *CAS9* and sgRNA. Efficiency increased to 81% after deleting the NHEJ-associated genes *KU70* and *LIG4*, suggesting that this DNA-repair pathway plays a major role in *C*. *lusitaniae*.

In 2017, Lombardi and colleagues [39] implemented a plasmid-based CRISPR–Cas9 technology in *C*. *parapsilosis*, in which the episomal pRIBO vector harbors the *NAT* marker, *CAS9* expressed from the *C*. *parapsilosis CpTEF1* promoter, and an sgRNA expression cassette. The sgRNA is expressed from the *C*. *parapsilosis* RNA pol II promoter *GAPDH* and flanked by a hammerhead (HH) and HDV ribozymes. Cotransformation of the replicating plasmid with a short donor DNA containing two in-frame stop codons flanked with 40-bp homology arms allowed for high-efficiency gene disruption. Despite variation in efficiency across strains, this method offers the possibility to edit any genetic background (e.g., clinical isolates). The plasmid is easily lost in the absence of selection, allowing sequential gene editing in the same genetic background. Zoppo and colleagues [40] showed that the system can also be used for gene editing in the sibling species *Candida orthopsilosis*. This system was recently improved by replacing the HH ribozyme with a tRNA molecule, which is recognized and cleaved by the yeast ribonuclease Z [29] (Fig 2A). This streamlined the cloning process, in that only the 20 bp guide region needs to be replaced to target a new gene. The editing system functions in *C*. *parapsilosis*, *C*. *orthopsilosis*, and *Candida metapsilosis* [29,41]. At the same time, a different plasmid was constructed that can be used in gene editing in *C*. *tropicalis* [29]. Notably, adaptation to *C*. *tropicalis* required not only changing the regulatory elements driving the expression of *CAS9*, *NAT*, and sgRNA—which were derived from *Candida dubliniensis*, *Ashbya gossypii*, and *Meyerozyma guilliermondii*, respectively—but also using a different autonomously replicating sequence, *ARS2* from *C*. *albicans* [29,42] (Fig 2A).

CRISPR–Cas9 can also be used in *C*. *glabrata*, a species that is not part of the CTG clade and is more closely related to *S*. *cerevisiae* than to *C*. *albicans*. Enkler and colleagues [19] transformed a triple auxotrophic strain (*his3*, *trp1* and *leu2*) of *C*. *glabrata* with a *LEU2* plasmid expressing the sgRNA and a *TRP1* plasmid expressing Cas9. By exploiting NHEJ—the preferential DNA-repair mechanism in this species—this strategy allowed up to 100% efficient *ADE2* gene disruption when using *C*. *glabrata CYC1* and *RNAH1* promoters to express *CAS9* and the sgRNA, respectively. Although 500-bp homology arms are usually recommended for HDR in *C*. *glabrata*, this method was used successfully to insert either short (57 bp) or large (1,202 bp) cassettes by HDR using only 20-bp or 200-bp homology arms, though 200-bp homology arms performed better for the small insert [43]. Vyas and colleagues [30] also generated an editing system in which *CAS9*, sgRNA, and *NAT* sequences are harbored on an episomal plasmid that can be used also in prototrophic clinical isolates of *C*. *glabrata* (Fig 2B). Transformation of the cells resulted in *ade2* mutants both with and without a repair template designed to introduce premature stop codons in the gene. Recently, Maroc and colleagues described a CRISPR vector containing the sgRNA cassette and expressing *CAS9* under the control of the *MET3* promoter, which can be used to induce mutations by either NHEJ or HDR in *C*. *glabrata* [44]. Since Cas9 expression is inducible, the authors foresee that this system may also be used for identifying essential genes in this asexual yeast.

As an alternative to expressing *CAS9* and the sgRNA in the cell, Grahl and colleagues [25] assembled Cas9 and the specific crRNA:tracrRNA duplex RNA in vitro to form an RNP (Fig 2C). The RNP was then transformed into *C*. *lusitaniae*, *C*. *auris*, and *C*. *glabrata* to induce gene deletion by homologous recombination of a selection marker flanked by 0.5–1 kb homology arms. Efficiencies ranged from 60% to 70%. However, this strategy does not allow marker recycling.

The implementation of the CRISPR–Cas9 technology in *Candida* species boosted our ability to decipher cellular signaling associated with virulence and the underlying genetic interactions. CRISPR–Cas9 used in combination with *C*. *albicans* mating-competent haploids allowed the investigation of epistatic relationships between members of gene families involved in fungal virulence (e.g., adhesins and efflux pumps) by generating libraries of homozygous double gene-deletion mutants [36]. This technology also streamlined the process of linking specific genes or SNPs (either heterozygous or homozygous) to relevant phenotypes in *Candida* species (e.g., resistance or sensitivity to drugs or cell-wall stressors [41,45–47].

### Aspergillus fumigatus

Gene manipulation in *A*. *fumigatus* is hampered by low efficiency of HDR (3%–5%), and NHEJ-impaired strains and 1-kb homology arms are traditionally used for site-specific integration [48]. Moreover, a reliable strategy of sexual reproduction in *A*. *fumigatus* has not been established, making mutagenesis of multiple genes by sexual crossing a challenging task, especially in clinical isolates. The first CRISPR–Cas9 method developed for *A*. *fumigatus* exploited NHEJ for gene disruption of the *pks* gene—involved in melanin production—by randomly integrating a cassette containing the hygromycin marker (*HPH*), the sgRNA expressed from the *S*. *cerevisiae SNR52* promoter, and the human optimized *CAS9* gene expressed from a *TEF1* promoter in the genome [16] (Fig 3A). Albino mutants were obtained with a 25%–53% frequency. Interestingly, unintended NHEJ-mediated integration of the transforming DNA to the Cas9 cut site was often observed. Notably, *CAS9* expression in the absence of a sgRNA did not alter growth rate or virulence in a murine model.

The following year, Zhang and colleagues [49] exploited MMEJ to efficiently (95%–100%) integrate desired sequences in-frame into a target gene via very short (approximately 35-bp) homology arms. They used strains constitutively expressing a human optimized *CAS9* gene from the fungal *GPDA* promoter on an autonomously replicating plasmid with a *PYR4* selectable marker. The sgRNA targeting the gene of interest, whose expression is driven by the native *A*. *fumigatus* RNA pol III *U6* promoter, can either be transformed as a linear cassette or directly provided to the cells as an RNA molecule following in vitro transcription. An *HPH* cassette flanked by microhomology regions can then be targeted to the cleavage site (Fig 3B). The same approach can be used to insert a green fluorescent protein (GFP) tag (although the selection for positive transformants is based on the random integration of the *HPH* cassette) and to create multiple mutants. Notably, this method can be used in clinical isolates [49].

In line with other *Aspergillus* species, Weber and colleagues [50] used the RNA pol II *GPDA* promoter to drive the expression of a HH–HDV ribozyme-flanked sgRNA. The authors adopted an ingenious split-marker approach to induce a single-nucleotide deletion in a NHEJ-defective strain containing an integrated tetracycline-inducible *CAS9*. An integrating plasmid harbors the sgRNA expression cassette along with the two halves of the split marker (the pyrithiamine resistance cassette (*PTRA*) of *A*. *oryzae*) interrupted by the same guide and PAM sequence that is being targeted in the genome. CRISPR–Cas9 mediated cleavage of the native target occurs in parallel with the cleavage of the “twin site” on the split marker, resulting in the reconstitution of the functional selection marker. The desired mutation can then be introduced by HDR with a donor DNA. Although inducible expression of *CAS9* lowers the risks of off-target mutations, it still remains in the genome.

Al Abdallah and colleagues [26] first used the RNP complex in conjunction with MMEJ for gene deletion in *A*. *fumigatus*. By using two sgRNAs to target Cas9-induced DSBs upstream and downstream of the *PKSP* gene and an *HPH* marker flanked with 35–50 bp homology arms as donor DNA, gene deletion occurred with an efficiency of >90% in both NHEJ-competent and NHEJ-defective strains. Although applicable to virtually any *A*. *fumigatus* isolates, this approach does not allow marker recycling, because the target gene is replaced by the *HPH* marker. Umeyema and colleagues [51] used a similar strategy to introduce two single point mutations previously identified in clinical isolates in the *CYP51A* gene to assess their role in azole resistance. In this case, the donor DNA was the full gene sequence containing the desired mutation(s), fused to the *HPH* marker. Therefore, despite several improvements, CRISPR–Cas9 in *A*. *fumigatus* still suffers from the absence of methods for marker recycling.

Single-base CRISPR–Cas9 editing has been used to gain further insights into the emerging phenomenon of azole resistance in *A*. *fumigatus*. The association of amino acid substitutions with azole resistance (e.g., Gly138Ser in Cyp51A [51]) also led to the discovery of a non-cyp51A mediated resistance mechanism relying on a mutated version of the *SVF1* gene [52].

### *Cryptococcus* species

Site-directed mutagenesis has been challenging in Basidiomycetes such as pathogenic *Cryptococcus* species for a long time. The main target species are *C. neoformans* (formerly known as *C*. *neoformans* var. *grubii*, serotype A), *C*. *deneoformans* (formerly known as *Cr*. *neoformans* var. *neoformans*, serotype D), and *C*. *gattii* [53]. Chemical transformation is not effective in these yeasts, and exogenous DNA is usually introduced into the cells by either biolistic transformation or electroporation. However, electroporated cells are unstable, and the efficiency of HDR ranges from 0.00001% to 0.001% [54,55]. By contrast, HDR following biolistic transformation rises to 1%–10%, and transformed cells are more stable [54], making this technique the gold standard for gene targeting in *Cryptococcus*. Unfortunately, biolistic transformation requires expensive equipment. In addition, although NHEJ-defective mutants have higher frequencies of HDR, their reduced virulence prevents their use in pathogenesis studies [56,57].

The implementation of CRISPR–Cas9 in *Cryptococcus* had a dramatic effect on increasing the efficiency of HDR, so much so that, in the last two years, electroporation is again being used for genetic manipulation. In the system developed by Wang and colleagues [17] for *C*. *deneoformans*, the sgRNA is expressed from a native cryptococcal RNA pol III *U6* promoter, and a *C*. *neoformans ACT1* promoter drives the expression of a human codon-optimized *CAS9* gene. Notably, electroporation of linearized URA5 vectors harboring the cassettes in a ura-strain resulted in 82%–88% pink auxotrophic colonies when *ADE2* was targeted by NHEJ (or NHEJ-equivalent) repair. Cotransformation with a 1-kb donor DNA also allowed a single codon change mediated by HDR. The *ADE2* gene was also disrupted by replacing with the hygromycin marker *HYGB* flanked by 1-kb homology arms on each side, although HDR occurred only in 8 out of 20 pink transformants, suggesting that the NHEJ pathway may be prevalent. Although the vectors were not integrated in the genome, the cassettes were stable in *C*. *deneoformans* even in the absence of selective pressure, increasing the risk of off-target effects and making gene complementation virtually impossible (the newly introduced wild-type (WT) copy of the disrupted gene will be recognized and cleaved).

In order to fix this problem, Wang and colleagues [17] further developed a “suicide” system that allows for the elimination of CRISPR components (Fig 3C). In this design, homology arms flank the *URA5* marker on the vector, driving its integration by HDR into the cleaved target gene. As the CRISPR and sgRNA elements are located on one side of the homology arm, they may be resolved by double crossover in HDR and eventually degraded. When put to the test, the “suicide” system efficiently disrupted the gene of interest and roughly 50% of the mutants lost the CRISPR elements, thus allowing subsequent complementation of the disruption.

Arras and colleagues [58] used biolistic transformation to integrate a *HYGB* cassette containing *S*. *pyogenes CAS9* expressed from a native *TEF1* promoter into the *C*. *neoformans* H99 Safe Haven region on chromosome 1 (Fig 3E). This strain can be subsequently transformed with a vector containing the G418 resistance marker *NEO* and expressing the sgRNA from the *C*. *neoformans* RNA pol II *ACT1* promoter, flanked with HH and HDV ribozymes [59].

Cotransformation of the vector transiently expressing the sgRNA and a cassette carrying approximately 1-kb homology arms on both sides led to the HDR-mediated gene disruption with an efficiency of 70%. Despite the concerns about the stable expression of Cas9, this system simplifies genetic manipulation in that it requires only the generation of the sgRNA vector and donor DNA constructs for targeting a gene of interest.

In 2018, Fan and Lin [60] developed TRACE (transient CRISPR–Cas9 coupled with electroporation) for both *C*. *neoformans* and *C*. *deneoformans*. Cryptococcal cells are electroporated with two linear cassettes with no selectable markers, in which *CAS9* and the sgRNA are under the control of native *C*. *neoformans* regulatory elements (*GPD1* promoter for the human optimized *CAS9* gene and *U6* promoter for the sgRNA targeting *ADE2*), together with a *NAT* cassette carrying 1-kb homology arms. Pink transformants were obtained with a 50%–90% efficiency depending on the strain, although further analyses showed that not all of the colonies were consistent with HDR-mediated integration of the cassette into *ADE2*. Moreover, both *CAS9* and the sgRNA were found randomly integrated into the genome in some of the transformants. Lowering the amount of Cas9 and sgRNA provided reduced the frequency of the unwanted integration events. TRACE can also be used to simultaneously target members of a gene family and to ectopically complement a mutated gene by introducing a WT copy into the Cas9-cleaved Safe Haven region (SH2).

Two additional approaches for *C*. *neoformans* and *C*. *deneoformans* were published by Wang in 2018 [27] (Fig 3D). Both methods rely on the simultaneous use of two guides, targeting the 5’ and the 3’ of the gene of interest, in an attempt to facilitate deletion by HDR with a *NAT* cassette carrying 800 to 900 bp homology arms. In the first approach, two plasmid vectors are transformed, each containing a *S*. *pyogenes CAS9* and one of the two sgRNAs expressed from *C*. *neoformans TEF1* and *U6* promoters. In the second approach, CRISPR elements are provided to the cells via electroporation as an RNP complex. Although more transformants were obtained using the plasmids, the RNP method remains a valuable approach, as it potentially allows the implementation of CRISPR–Cas9 in species for which expression cassettes are not available yet (e.g., *C*. *gattii* and *C*. *deuterogattii*).

### Mucorales species

The generation of stable gene replacements by HDR is a challenging process in Mucorales, because plasmids tend to not integrate regardless of the size of the homology arms. The first CRISPR–Cas9 method available uses the RNP approach to generate stable mutants in *Mucor circinelloides* [28]. Different to other RNP methods, assembly of Cas9 with the sgRNA occurs in vivo, inside the fungal protoplasts. The Cas9-induced DSB resulted in 100% efficiency for both gene disruption by NHEJ and gene deletion by HDR-mediated integration of a *PYRG* cassette flanked by homology arms. Notably, NHEJ led to an unexpected >2.3kb-long deletion upstream of the PAM in the resulting mutants, possibly causing unwanted effects (i.e., the gene adjacent to the targeted one was also affected by the deletion). The HDR pathway is therefore preferable, despite resulting in the stable integration of the section marker. Despite very low transformation efficiency, this method provides stable mutants, streamlining the genetic manipulation of these genetically intractable fungi. Earlier this year, Wang and colleagues demonstrated that a CRISPR–Cas9 plasmid formerly developed for *Cryptococcus* species could be used for NHEJ-mediated gene disruption in *Rhizopus delemar* [61]. The implementation of CRISPR–Cas9 technology in Mucorales provides a much needed window into the biology of these emerging opportunistic pathogens for which robust genetic tools are lacking.

## Perspectives and future directions

Genetic manipulation of fungal pathogens has been a milestone in deepening our understanding of virulence and host–pathogen interactions. The generation of single-gene mutant libraries (e.g., in *C*. *neoformans* [62], *C*. *albicans* [63], and *C*. *parapsilosis* [64], as well as other approaches like promoter replacement [65], transposon mutagenesis to study gene essentiality [66], and heterozygous libraries generated for haploinsufficiency-based genetic interaction studies in *C*. *albicans* [67] allowed the dissection of the molecular players of fungal biology and pathogenicity.

CRISPR–Cas9 methods are now available for most of the major fungal species causing infections, facilitating genetic engineering by increasing the rate of homologous recombination as never seen before. Unique guide sequences are available to target >90% of the annotated *ORFs* in both *C*. *albicans* and *C*. *glabrata* genomes [18,30]. Different genetic changes can be introduced (e.g., deletion, single-nucleotide mutation, stop codon insertion, barcoding, fluorescent tagging, etc.) in coding as well as noncoding regions. Ongoing research in model eukaryotic organisms like *S*. *cerevisiae* keeps expanding the boundaries of potential CRISPR applications [11,68]. An interesting refinement is the possibility of engineering heterozygous mutations in diploid species, which allows the study of heterozygous variants or essential genes. This was first achieved in mammalian cell lines using mixed repair templates carrying a different set of mutations or, alternatively, by exploiting the cut-to-mutation distance (the efficiency of mutation incorporation rapidly falls with increasing distance from the Cas9 cleavage site) [69]. Recently, heterozygous mutations were introduced in *C*. *parapsilosis* and *C*. *orthopsilosis* by applying the same principle [29,41].

Larger chromosomal deletions (>30 kb) and chromosome fusion have been achieved in *S*. *cerevisiae* through NHEJ repair between endpoints of the DNA breaks, which can facilitate the study of gene clusters or minimal genome research, as well as the study of replication, recombination, and segregation [70,71,72]. In principle, this may open up the possibility to generate CRISPR–Cas9–induced genome rearrangements in *C*. *albicans*, in which specific whole-chromosome aneuploidies have been associated with drug resistance and/or cross-adaptation to different drugs [73]. In *S*. *cerevisiae*, the introduction of a DSB by Cas9 can result in extensive loss of heterozygosity (LOH) events consistent with HDR [74]. Whereas this needs to be taken into account when manipulating polyploid and highly heterozygous genomes, it could also be exploited to study the impact of LOH on *C*. *albicans* virulence. In fact, exposure to the mammalian host increases the frequency of LOH in *C*. *albicans*, likely selecting for specific mutations. In addition, LOH is associated with reduced drug susceptibility in strains sequentially isolated from the same patient [75].

Although most of our knowledge on microbial pathogenesis was built on extensive analyses of one or few laboratory strains, it is becoming increasingly clear that genotype–phenotype correlation should be validated using multiple isolates. An example is the regulatory network controlling biofilm production and hyphal formation in *C*. *albicans*; circuit diversification appears to be widespread in different isolates, as attested by the fact that loss of function of the same transcription factor can have a different impact on the network depending on the strain [76]. Many of the CRISPR–Cas9 systems developed for pathogenic fungi are applicable to any genetic background, thus allowing the generation of mutations in different clinical isolates and a more robust validation of gene function.

Targeting gene families is also possible by exploiting the homology within coding regions of paralogs. For example, Wijsman used 11 unique guide sequences to delete 21 hexose transporters in *S*. *cerevisiae*, in only 3 transformation steps [77]. Multiplexing is time-saving and triggers less chromosomal rearrangements than the Loxp/Cre system, ensuring high fidelity when addressing extensive genome editing strategies [77]. CRISPR–Cas9 based gene-drive strategies had been developed in *S*. *cerevisiae* and *C*. *albicans* [36,37,78], in which the selfish element itself is used as donor DNA and replaces the WT allele after Cas9 cleavage. In diploid organisms with sexual reproduction, all progeny cells will inherit the gene-drive, bypassing mendelian inheritance [37].

CRISPR–Cas9 made the construction of genome-scale mutant libraries easier than ever before, thus streamlining downstream applications such as genome-wide variant engineering [79] or gene interaction screens [80], which are useful in identifying fungal-specific genes involved in essential biological processes that could represent potential new therapeutic targets. Finally, directed evolution studies are another perspective that can be achieved for example by a CRISPR-guided nickase fused to an error-prone DNA polymerase, dramatically increasing the mutation rate [81]. Such strategies favoring directed evolution could facilitate the identification of new variant genotypes with successful virulence or antifungal resistance traits.

Besides Cas9, at least 10 different Cas enzymes have been described, differing in structure, PAM recognition sites, or type of cleavage mechanisms, allowing new targeting options [82]. Examples are eSpCas9, SpCas9-HF1 and HypaCas9 (engineered variants of Cas9 with a higher specificity [83–85]), Cas12a (less off-target effect; PAM = TTN; appropriate for low GC-rich regions) [86], and xCas9 (broadest PAM compatibility; PAM = NG, GAA and GAT)[87]. On the other hand, developing CRISPR base editors paved the way for genome editing without introducing DSB DNA-breaks by inducing gene conversions such as C to T, G to A [88], or A-T to G-C [89]. Anzalone and colleagues further improved this strategy by combining a catalytically impaired Cas9 (only able to nick one DNA strand) with a reverse transcriptase; the complex is driven to the target site by a prime editing guide RNA (pegRNA) that also encodes the edit [90]. This method enables precise targeted insertions and deletions and all 12 possible classes of point mutations (including transversion mutations) without inducing DSBs, therefore minimizing side effects, and at the same time not relying on HDR with a donor DNA to edit the genome. While this is particularly relevant in mammalian cells, it could also be applied to fungi in which the efficiency of HDR remains low, even following a Cas9-induced DSB.

One more step has also been reached by editing RNAs using Cas13 enzymes, making nonpermanent changes of the genome possible [91].

Nonediting applications using Cas9 have also emerged over the years [92]. Fusion of Cas proteins with reporters allows live-cell imaging of DNA and RNA [93]. A catalytically dead variant with no nuclease activity (dCas9) but retaining its binding activity has been used to regulate gene expression in *S*. *cerevisiae*, allowing either activation (CRISPRa)—when fused to a transcriptional activator—or interference (CRISPRi), when fused to a repressor [94]. Similar systems have recently been developed for *C*. *albicans*, in which comprehensive genetic interaction maps could not previously be built using RNAi-based genetic knockdown, because RNAi is not effective in this species. The faster and cheaper genetic manipulation of fungal genome and expression facilitated by CRISPR–Cas9 is likely to have a profound impact on medical mycology. Loss of function, gene activation, and gene inhibition strategies can be scaled up as CRISPR–Cas9 libraries [95–97], and large-scale library screens can facilitate the identification of new targets for antifungal therapy, of which there is a desperate need [98].

The use of RNA–Cas9 complexes is particularly important in species refractory to genetic manipulation, making easier and more timely the study of emergent and multiresistant fungi. Although not yet applied to medical mycology, the diagnostics field already started harnessing the potential of CRISPR–Cas9. Myrhvold and colleagues, developed a Cas13-based nucleic acid detection platform, Specific Highly sensitive Enzymatic Reporter unLOCKing (SHERLOCK), coupled with detection by a lateral flow device as a point-of-care test to diagnose Zika and Dengue virus in clinical samples [99]. The extension of this technique to the high sensitivity and specificity detection of fungal DNA could radically improve the diagnosis of fungal infections, especially in low resource settings [100]. CRISPR–Cas9 and Next Generation Sequencing were combined in Finding Low Abundance Sequences by Hybridization (FLASH), a new method for multiplex detection of antimicrobial resistance in clinical samples of gram-positive bacteria and *Plasmodium falciparum* [101].

The potential applications of CRISPR-based methods developed in either model fungi (like *S*. *cerevisiae)* or bacteria, viruses, and mammalian cells to the study of fungal pathogenesis are summarized in Table 1.

**Table 1:** Potential translation of CRISPR-based methods developed for model organisms to the study of fungal pathogenesis.

Method	Developed in	Potential application to fungal pathogens	Refs.
CRISPR-mediated large chromosomal deletions and fusions	*S*. *cerevisiae*	Study of the correlation existing between large chromosomal rearrangements and virulence and drug resistance (e.g., aneuploidy and LOH in *C*. *albicans*, aneuploidy in *C*. *neoformans*)	[70–72]
Directed evolution through CRISPR-guided DNA polymerase	*Escherichia coli*	Identification of new variant genotypes with virulence or antifungal resistance traits	[81]
CRISPR prime editing	Human cells	Precise DSB-free editing in species refractory to HDR	[90]
Cas13-based nucleic acid detection platform (e.g., SHERLOCK)	Zika and Dengue viruses	Rapid diagnosis of fungal infections in low resource settings based on the development of highly sensitive (attomolar range) kits for detection of fungal DNA	[99]
CRISPR–Cas9 based enrichment of sequences for NGS (e.g., FLASH)	Gram-positive bacteria, *P*. *falciparum*	Enrichment of fungal sequences associated with a trait of interest (e.g., antifungal resistance) prior to NGS, potentially resulting in antimicrobial resistance profiling directly from the patient sample	[101]

CRISPR, clustered regularly interspaced short palindromic repeats; DSB, double-strand break; FLASH, Finding Low Abundance Sequences by Hybridization; HDR, homology-directed repair; LOH, loss of heterozygosity; NGS, next generation sequencing; SHERLOCK, Specific Highly Sensitive Enzymatic Reporter UnLOCKing

## Conclusions

This review provides an updated overview of the state of the art of CRISPR–Cas9 genome editing in pathogenic fungi to both researchers and medical mycologists interested in using this technology. Though we focused on major fungal pathogens encountered in the clinical setting [102], CRISPR–Cas9 methods have also been developed in other human fungal pathogens not discussed here, such as *Malassezia* [103], *Fusarium* [104], and the dimorphic fungus *Blastomyces dermatitidis* [105]. The effectiveness and flexibility of CRISPR–Cas9 have the potential to have a dramatic impact on the field of medical mycology, both in the lab—by boosting genetic manipulation for studying fungal biology and pathogenesis, dissecting the role of virulence factors, and investigating new potential drug targets and host–pathogen interaction—and at the bedside, by improving the diagnosis of fungal infections and the monitoring of antifungal resistance [4]. Moreover, further applications currently developed in model fungi may be applied to fungal pathogens in the near future for studying host–fungi interactions or tackling antifungal resistance [106].
